# Using of geographic information systems (GIS) to determine the suitable site for collecting agricultural residues

**DOI:** 10.1038/s41598-022-18850-0

**Published:** 2022-08-26

**Authors:** El-Sayed G. Khater, Samir A. Ali, Mohamed T. Afify, Magdy A. Bayomy, Rasha S. Abbas

**Affiliations:** 1 Agricultural and Biosystems Engineering Department, Faculty of Agriculture, Benha University, P.O. Box 13736, Banha, Egypt; 2 Institute of Agricultural Engineering Research, Agriculture Research Center, Doki, Giza, Egypt

**Keywords:** Ecology, Environmental sciences, Engineering

## Abstract

The main aim of this study is to use the Geographic Information Systems (GIS) techniques to determine the optimum site to collect the residues in order to reduce cost and increase the benefits. To achieve these three scenarios were studied to reach the best collection sites for recycling rice straw in Sinbilawin center. The results indicate that the first scenario: The result was forty (40) collection sites in this status the cost of transfer will be very high because the collecting starts from inside village to the 40 sites and transfer to main sites to recycle operation. The second scenario: The total lengths of roads are not much then the cost of transfer is low and save time and efforts. The third scenario: The result was five collecting sites. It was shortest length and lowest cost. Transportation costs in the first scenario were difficult to calculate because of the difficulty to access a network of documented roads from satellite maps to use it with the GIS program. The total internal transport costs were 987,308.86 and 826,966.43 L.E (Egyptian pound, $ = 19.15 L.E) for second and third scenarios, respectively. The average transport costs per ton were 17 and 14 L.E/ton for the second and third scenarios, respectively. Also, the total lengths of roads were 817.62 and 615.65 km for the second and third scenarios, respectively.

## Introduction

Waste represents a big problem in whole world^1^. It is very dangerous on the environment and human health, in addition to diseases transfer, fire hazards, water pollution and economic losses^2,3^.

In Egypt, the total crop residues production of about 35 million tons per year^4^. Economic Affairs Sector^5^ reported that the summer season is the highest crop residues production as it reached 73%. Winter season production comes in the second as it reached 27%. The total area of rice crop in Egypt is 1,215,830 faddan (faddan = 0.42 hectare) and the production of rice is 4,817,964 tons. The average of productivity is 3.963 tons. The total cultivated rice area of Dakahlia is about 380,661 Fadden and the production of rice is 1,686,328 ton.

Rice straw is considered the most important crop residue in the world because it has in many uses such as feeds, composite and mulch for planting. Huge quantity of this straw is burn causing a great loss economically and environment pollution and other uses like ethanol, paper and fertilizes^6^.

In Egypt, 20% of rice straw used for ethanol, paper, fertilizes and fodders and the left part of straw is burn resulting emissions contribute to air pollution is called "Black Cloud"^7^.

Waste management is very important issue these days in the world. Unsuitable methods of handling these wastes could lead to economic and environmental problems^8^. The amount of waste increase by increasing population in the world. Environmental pollution is caused by organic waste. Organic wastes could be used in landfill, incineration, pyrolysis, biogas and composting processes. Biogas and compost processes provide economical valuable products and reduce the environmental impact^9^.

Geographic Information Systems (GIS) is defined as a computer software and hardware which sorted, manipulate, analyze and display spatial or geographically referenced data^10,11^. It makes maps and updates data GIS is very important information system used as a tool in whole processing system. GIS is used in solving several problems depending on data^12–15^.

Geographic Information Systems (GIS) is very good tool in handling data in many aspects such as optimum locations for transportation, locating new landfills and landfill monitoring and it saves time and costs; it has the capability of managing a large amount of data from different sources^16,17^. Geographic Information Systems (GIS) can offer various opportunities for improving the convenience and accuracy of spatial data, more productive analysis and improved data access^18^. Also, it could handle large amount of spatial data to be processed. It stores, retrieves, analyzes and displays information according their specifications. But it can be limited by existing sources of data required in sitting analysis^19–21^.

Due to gradually increasing of transporting costs of the crop residues from place to another to get benefit from it, this led to the most farmers to burn their crops residues, which result in emissions significantly contribute to the air pollution called the black cloud. Therefore, the main aim of this study is to use the Geographic Information Systems (GIS) to determine the optimum site for the residues collection in order to reduce cost and increase the benefits.

## Materials and methods

### Materials

#### Study area

**Figure 1:**
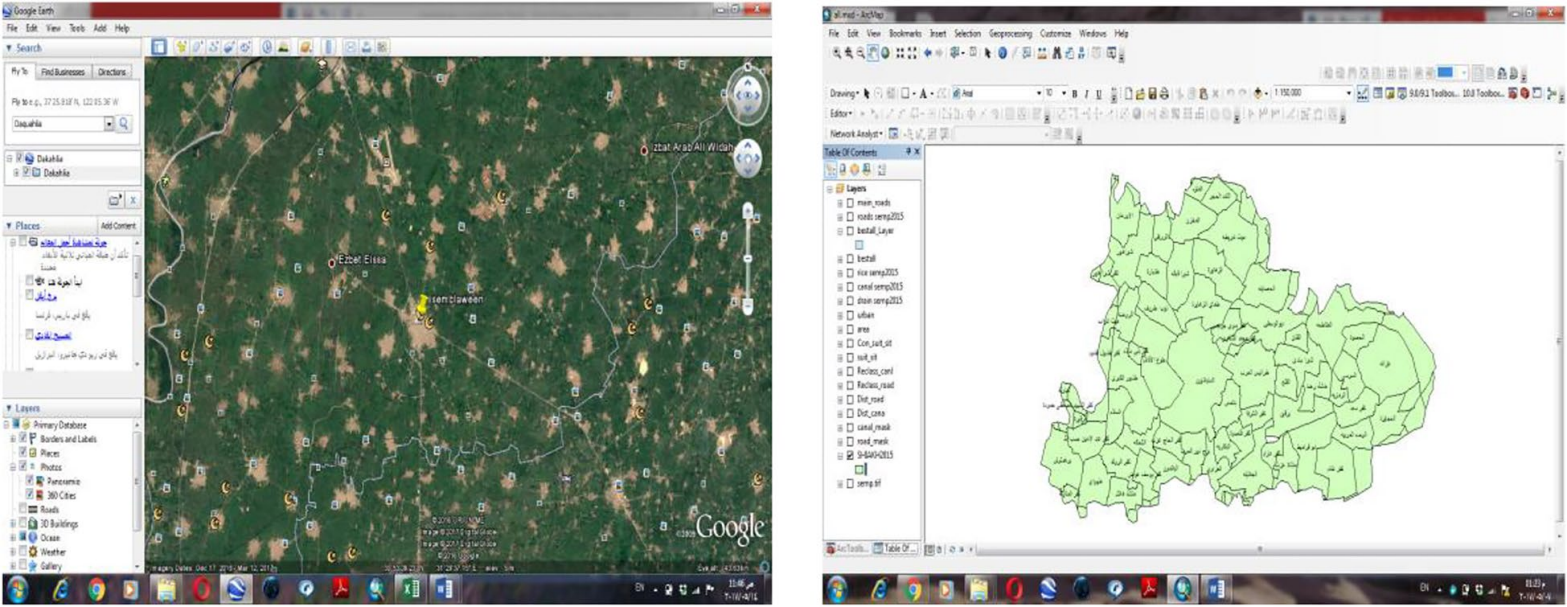
Map of the Sinbilawin city, 2015 (study area).

The Sinbilawin town is located southeast of Dakahleia Governorate, Egypt. It is bounded to the east by the Timai El-Amded city, west by the Aga city, north by the Mansoura city and to the south by the Diarb Negm city. The Sinbilawin lies between 31° 27′ 38.07″ E longitude and 30° 53′ 1.55″ N latitude (Google Earth) (Fig. 1). The total area of Sinbilawin town is about 304.5 km^2^ with total cultivated area of Sinbilawin is about 64,362.28 Faddens^5^. The Sinbilawin town is characterized a flat land.

#### Rice straw

**Figure 2:**
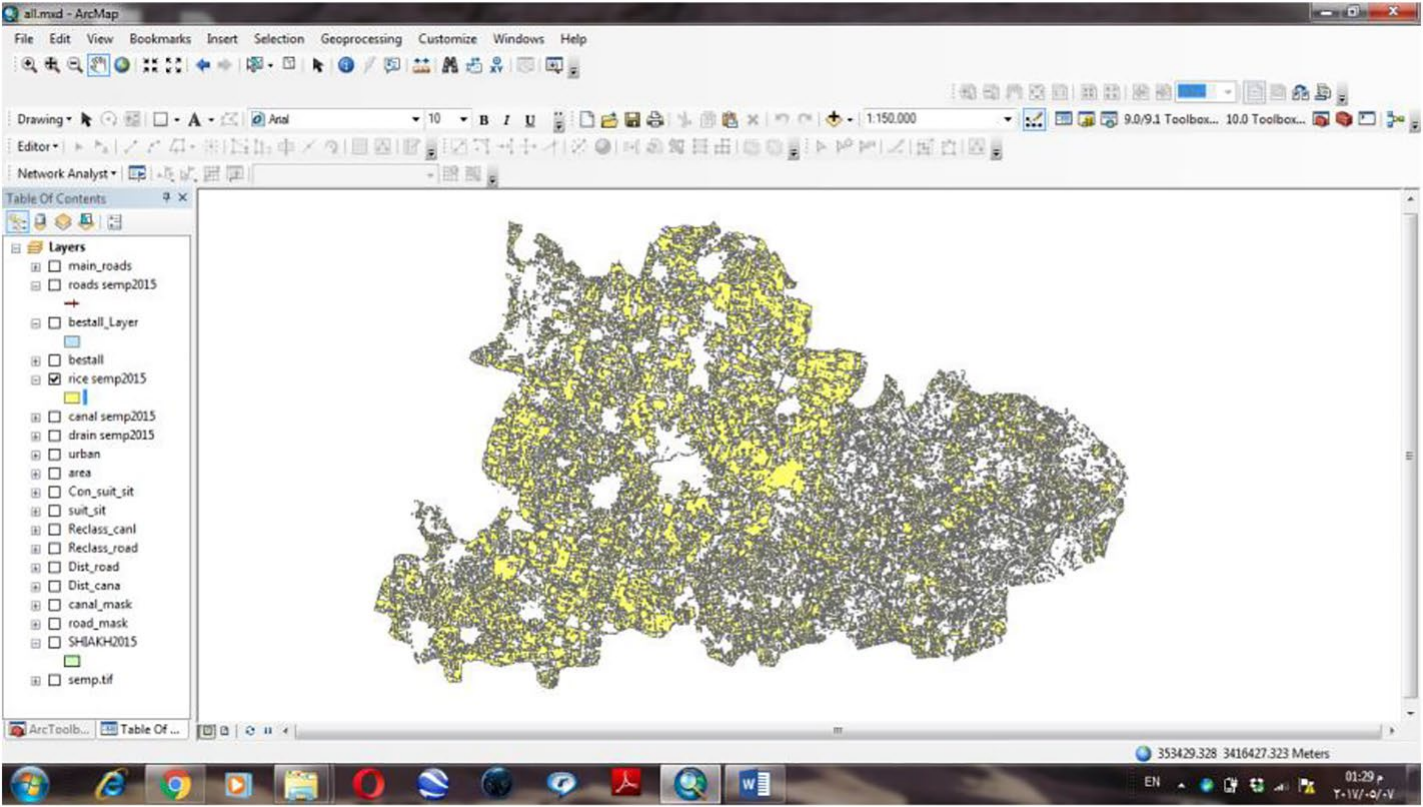
Rice area map.

The total area of rice crop in Egypt is 1,215,830 faddan and the production of rice is 4,817,964 tons. The average of productivity is 3.963 tons^5^. The total area of rice crop in Sinbilawin center is 34,078.12167 faddan and the production of rice straw is 148,376.1417 tons. The rice area map is shown in Fig. 2.

#### Data

**Figure 3:**
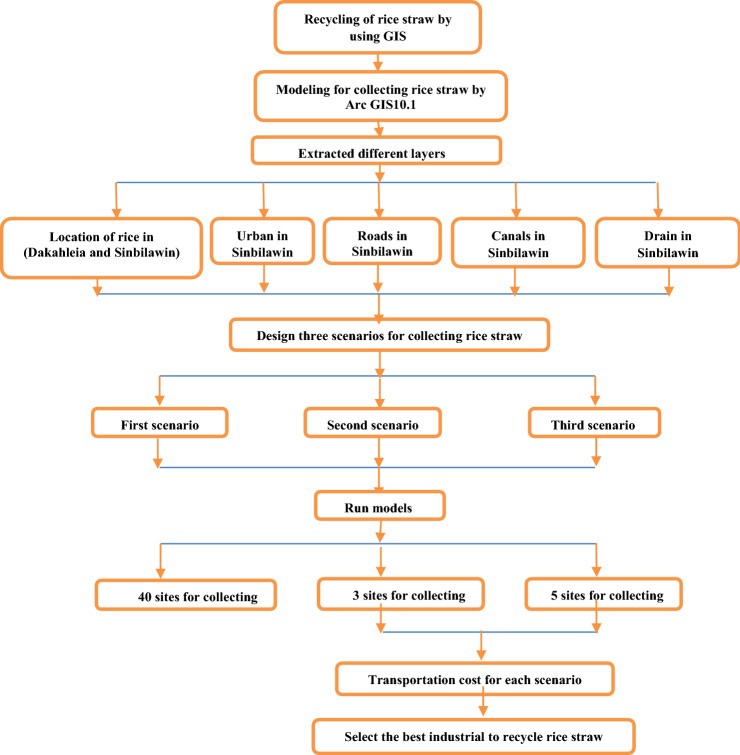
Flowchart of rice straw collecting from Sinbilawin center.

GIS is a powerful tool which used for computerized mapping and spatial analysis. GIS is used in many applications such as geology, protection, natural resource management, risk management, urban planning, transportation, and various aspects of modeling in the environment. Also, it is using for decision making^22^. In this study GIS is used to select the best site to be suggested to collect the rice straw as shown in flowchart of Fig. 3.

##### Software programs

- Google Earth program
  Google Earth combines the power of Google Search with satellite imagery, maps, Terrain and 3D buildings to put the world's geographic information at your fingertips. It displays satellite images of varying resolution of the Earth's surface, allowing users to see things like cities and houses looking perpendicularly down or at an oblique angle, with perspective^23^.
- Image Processing and Analysis Software (ENVI) program
  It has been used to separate layers from the satellite image as layer of road, layer of urban, layer of canal and layer of sites to the rice crop planting. ENVI 5.6.2 Classic is the ideal software for the visualization, analysis and presentation of all types of digital imagery. ENVI Classic’s complete image-processing package includes advanced, yet easy-to-use, spectral tools, geometric correction, terrain analysis, radar analysis, raster and vector GIS capabilities, extensive support for images from a wide variety of sources, and much more^24^.
- GIS program
  ArcGIS Desktop 10.1 will be using in the present study. It is the newest version of a popular GIS software which produced by ESRI. ArcGIS Desktop is comprised of a set of integrated applications. All figure numbers were created using GIS software.

#### Design a model for assembling rice straw

ArcGIS10.1 was selected in this study to design a model for selecting the suitable sites to collect rice straw amounts in Sinbilawin center. To achieve the former goal must be gotten the satellite images (landsat 8) for the province of Dakahleia and the Sinbilawin center. These images were called operation land imager (OLI). Thus, layers will be obtained from the satellite images such as water channels, drainages, urban areas, main and sub- roads, rice crop areas and sites. ENVI program has been used to separate layers and place it in a file which named (Shp. file) for easy insertion in ArcGIS10.1 program. In this present study, design a model will be done on the main layers which will be obtained from the satellite image as follows:

- Location and the administrative limits of Dakahleia Governorate and Sinbilawin center.
- The rice crop area and sites in Dakahleia governorate as the main layer.
- Layer of rice area and their sites in Sinbilawin center. Sinbilawin center was selected in the study because it is cultivated largest rice area in Dakahleia and Dakahleia biggest governorate cultivates rice.
- Layer of roads network in Sinbilawin center. The network of roads was included the main roads and submain to aggregation rice straw. Given the problems associated with transport cost, disposal, and issues that arise from inadequate agriculture crop residues management, the collect units become essential to be nearest of the network of road to facilitate the process of transportation and minimize cost.
- Layer of the urban locations in Sinbilawin center. Crop residues collection sites have an enormous impact on urban in general due to contamination and fires. This study proposes the collecting rice straw sites not be near of the urban, because it causes many health problems for the population.
- Layer of the canal locations in Sinbilawin center. Collecting rice straw sites must be nearest from the source of water as canal for safety, protect it from fire and important for any recycle operation.
- Layer of the drain locations in Sinbilawin center. Also, drain is important as the source of water but less than canal.

##### Arc GIS 10.1 to select the suitable sites for assembling rice straw

Three Scenarios were suggesting for completing the design of the modeling to select best sites for collecting rice straw. From the three scenarios wall be reached to the best collecting sites for rice straw in Sinbilawin center as follows:

- The first scenario: Modeling for Sinbilawin center
  In this case, modeling was running on the Sinbilawin center as the whole unit.
- The second scenario: Modeling for the village in Sinbilawin center.
  The Sinbilawin center consists of 97 villages and some other area surrounding. In this case, modeling was running on each village and each accessory in Sinbilawin center.
- The third scenario: Modeling for the best site in each village in Sinbilawin center.
  In this case, the modeling was running on each best site which located in each village (on the 97 sites in Sinbilawin center).

### Methods

To achieve the former objective in this study wall be done as follows:

- Location and the administrative limits of Dakahleia Governorate and Sinbilawin center were uploaded as map by Google earth program.
- The rice crop area and sites in Dakahleia governorate. The data of area and sites to rice crop in Dakahleia governorate were collected from the Ministry of Agricultural—Central Administration of Economy and Statistics as numerical data for each center in Dakahleia governorate. Map for Dakahleia governorate was obtained via satellite image from the Remote Sensing Authority.
- Rice production (ton) = Cultivated area(fed)*Average production (4.354 ton/fed)^5^.
- Total rice straw (ton) = Rice production (ton) / 2.5.

#### Satellite image layers

##### Areas and sites of satellite layers for rice in Sinbilawin center

Area and sites of rice crop in Sinbilawin center as the database were obtained and collected Extraction layer from the Ministry of Agricultural. Central Administration of Economy and Statistics as numerical data for each village. Sinbilawin map as layer of molding was obtained via satellite image from the Remote Sensing Authority. It was used with ArcGIS 10.1 software to inference the sites and area of rice crop in the Sinbilawin center villages.

##### Layer for the road network in Sinbilawin center

The network of roads is very important factor and effective for collecting rice straw. The network roads map as the layer was extracted from satellite image via the Remote Sensing Authority. It was used with ArcGIS 10.1 software to inference the main and sub roads in the Sinbilawin center.

##### Layer for the urban locations in Sinbilawin center

Crop residues collection sites have an enormous impact on urban general due to contamination, environmental pollution and fires, which are causing many health problems for the population. The urban map as the layer was extracted from satellite image via the Remote Sensing Authority. It was used with ArcGIS 10.1 software to appear all the urban sites in the Sinbilawin center.

##### Layer for the water source in Sinbilawin center

Rice straw collection sites must be nearest from the source of water as canal for safety and protect it from fire also water is very important for any recycle operation. The canal map as the layer was extracted from satellite image via the Remote Sensing Authority. It was used with ArcGIS 10.1 software to appear all source of water as canal in the Sinbilawin center.

##### Layer for the drain locations in Sinbilawin center

The drain is important as the source of water but less than canal. The drain map as the layer was extracted from satellite image via the Remote Sensing Authority. It was used with ArcGIS 10.1 software to appear all drain in the Sinbilawin center.

#### ArcGIS 10.1 to select the suitable sites for collecting rice straw

**Figure 4:**
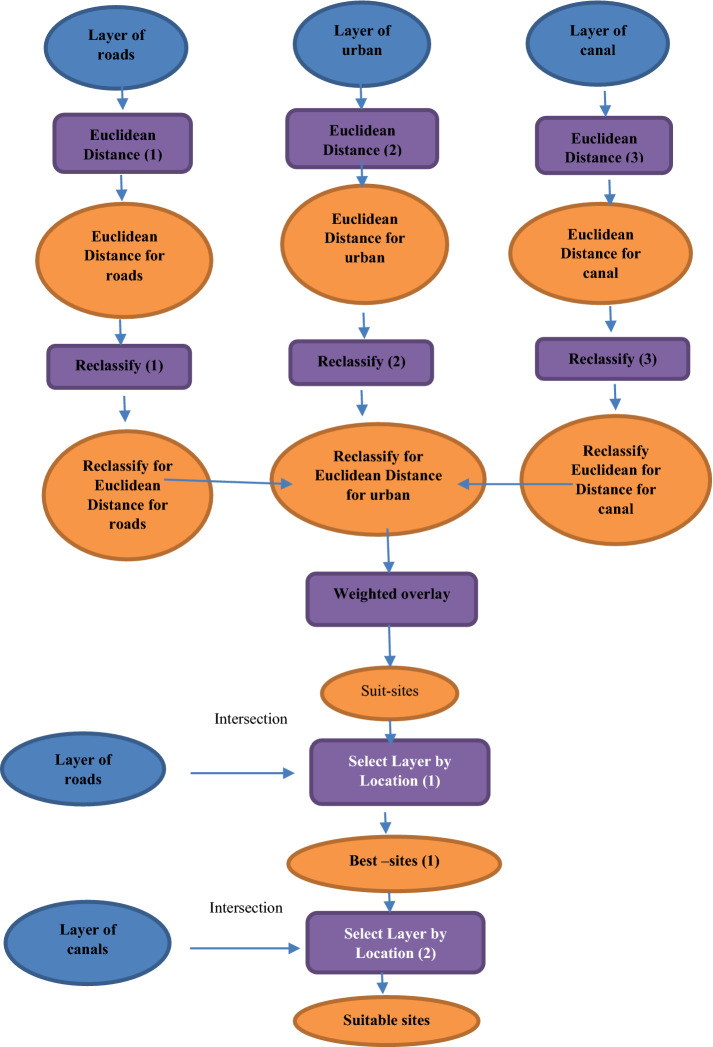
Short form for modeling to select suitable sites to assembly rice straw.

Modeling was designed as shown in Fig. 4 to apply with the three scenarios.

From the three scenarios shall be reached to the best collecting sites for recycling rice straw in Sinbilawin center as follows:

- The first scenario was running modeling for Sinbilawin center.
- The second scenario was running modeling for the village in it.
- The third scenario was running modeling for the best site in each village in it.

Different steps were running with modeling to select the best sites to assembly rice straw in Sinbilawin center: 1- Euclidean distance. 2- Reclassify (or changes). 3-Weighted overlay. Assuming common measurement scale and weights for each layer according to its importance as follows:—Roads 50%, Channels 40%, Urban 10% so that the total is 100%0.4- Select Layer by Location (Data Management). In this step, order of selecting layer sites was given through Arc tool box at ArcGIS10.1 for selecting sites through the Arc toolbox at ArcGIS10.1 software as follow: 1- Intersection with roads. 2- Intersection with canals water.

#### Total cost of collecting rice straw

Transportation for collecting crop residues is important factors because it affects the success or failure of crop residues utilization. GIS was used to determine suitable sites for collecting rice straw and converting it through given parameters as:

- Total length of road (km).
- Total weight of rice straw (ton).
- Speed of tractor in sub roads (30 km/h)
- Total time of transfer (h).

All experimental protocols were approved by Benha University Research Committee and all methods used in this study was carried out according to the guidelines regulations of Benha University. This work is approved by the ethic committee at Benha University.

## Results and discussions

### The suitable sites selection for collecting rice straw

In order to reach the goal of study, selecting the best site for rice straw collection to save time and cost in Sinbilawin center, three scenarios were following as follows:

#### First scenario

**Figure 5:**
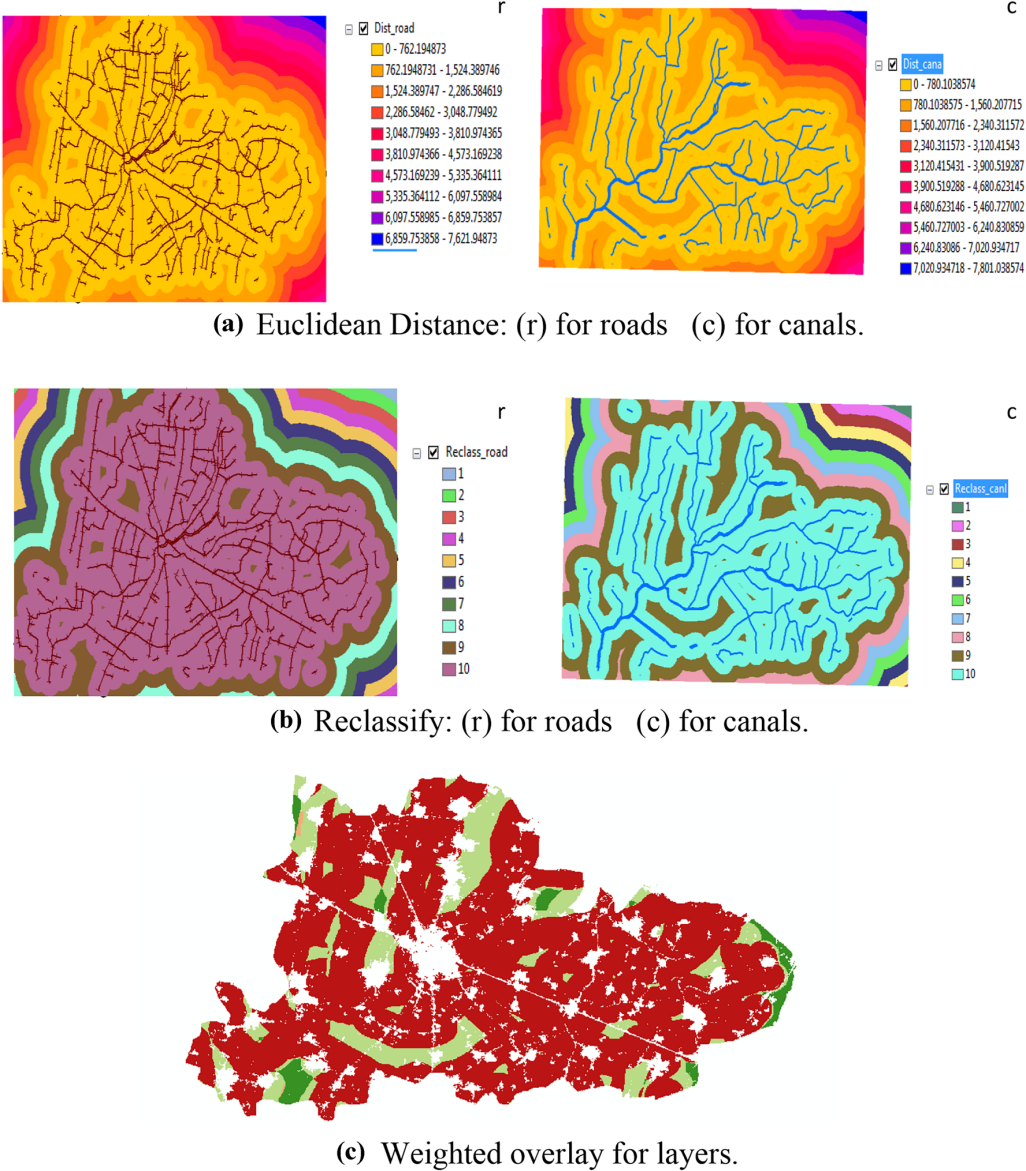
**(a)** Euclidean Distance: (r) for roads (c) for canals. **(b)** Reclassify: (r) for roads (c) for canals. **(c)** Weighted overlay for layers.

Euclidean Distance, reclassify and weighted overlay are shown in Fig. 5a–c. The Euclidean distance was determined as the nearest and furthest point from the road to the administrative limits Sinbilawin center. The result was represented by different colors and distances. The nearest distance was yellow color and 0 but the furthest distance was blue color and 7,621.94873 km. Also, the canal layer was represented by different colors and different distances. The nearest distance was yellow color and 0 but the furthest distance was blue color and 7,801.038574 km.

Reclassify the roads and canals layers which produced from the previous step. Also, it was classified the new layers to be equal interval. The final result was produced ten (10) different colors of categories.

Several layers were overlaid as the form of raster to collect it in one layer through the weighted score. This layer was collected between roads and canal which represented by amusement scale according important it (roads 50% and canal 50%). The final results were: Weights range from 0 to 10 and 0 means unsuitable while 10 means suitable.

**Figure 6:**
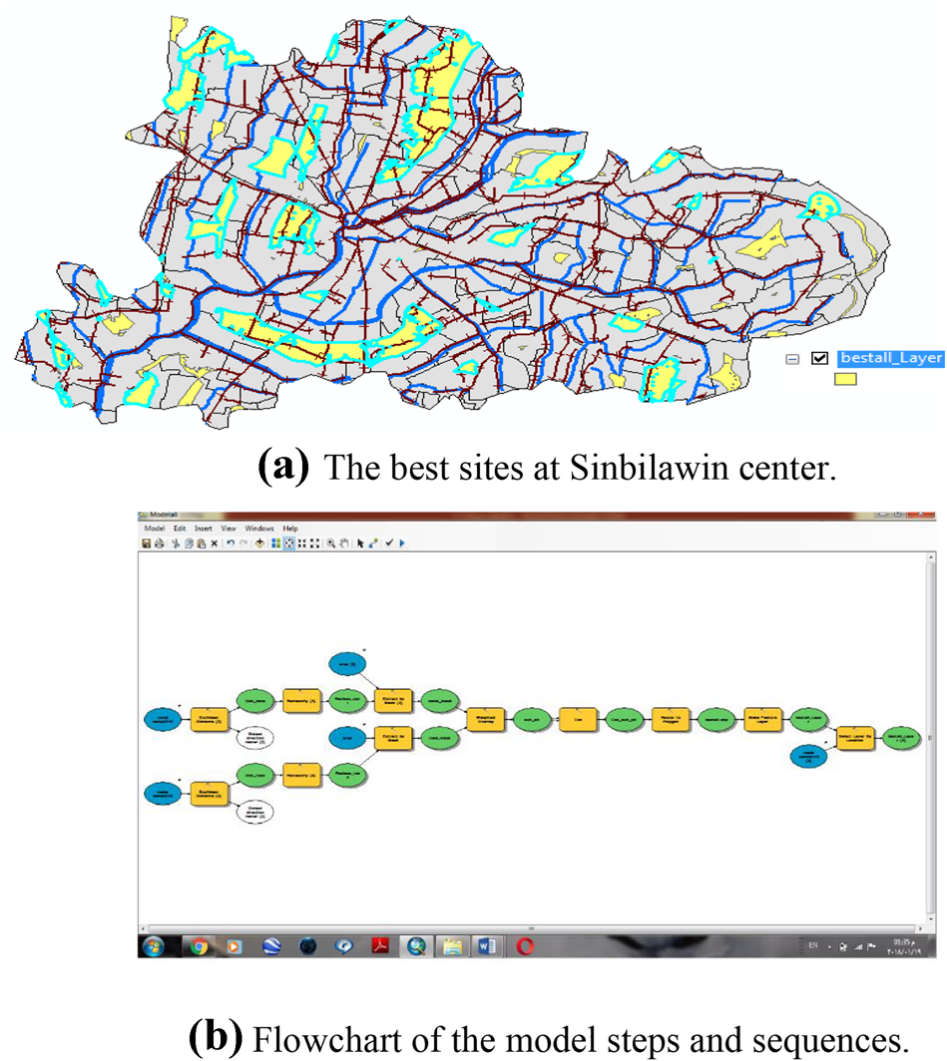
**(a)** The best sites at Sinbilawin center. **(b)** Flowchart of the model steps and sequences.

Finally result, 40 sites from 196 sites were selected for the collection and management of rice straw as Fig. 6a,b. Figure 6a shows the forty sites from 196 sites were selected for collecting and management of rice straw, while, Fig. 6b shows the flow chart of the model steps and sequences.

#### Second scenario

In this step, was extracted layer for 97 villages and other area surrounding in Sinbilawin center.

**Figure 7:**
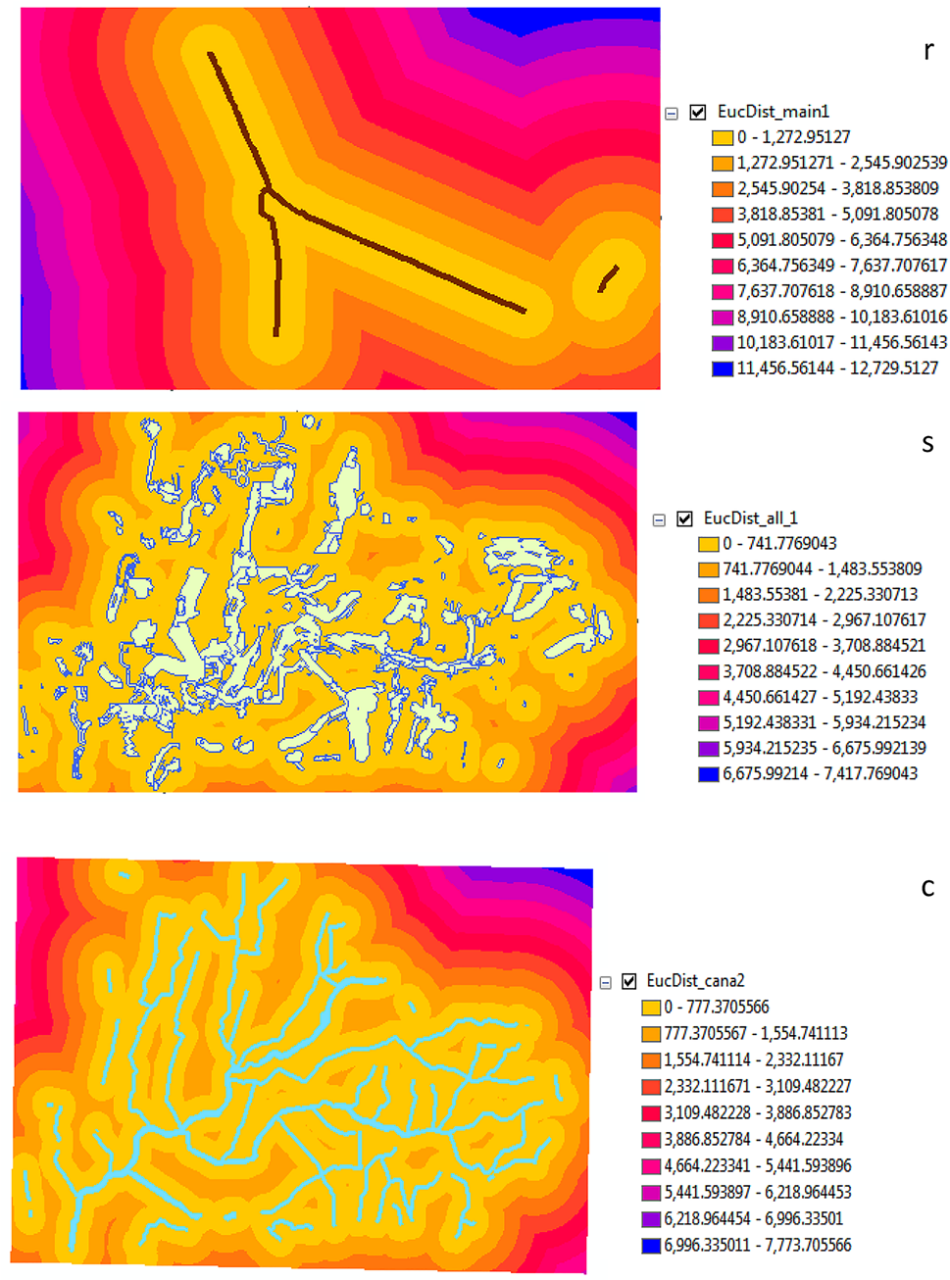
Euclidean distance: (r) for main road (s) for all sites (c) for canal.

Three layers were resulted from these step roads, canals and a new layer as Fig. 7. The nearest distance for roads was yellow color and 0 but the furthest distance was blue color and 12,729.5127. Also, the nearest distance for canals was yellow color and 0 but the furthest distance was blue color and 7,773.705566.

**Figure 8:**
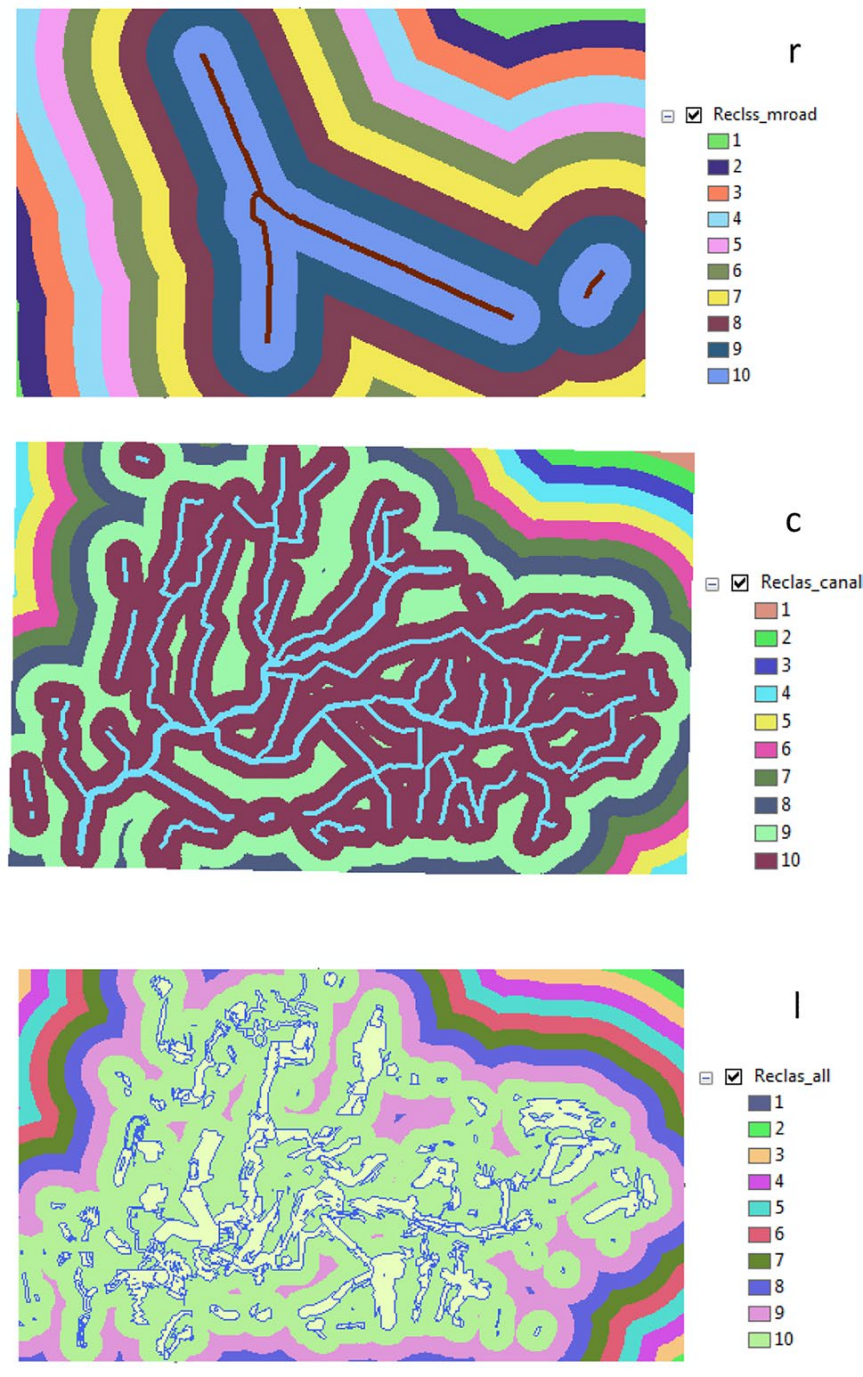
Reclassify: (r) for main road (c) for canals (l) for new layer.

From reclassification resulted three categories for canals, main roads and anew layer as Fig. 8.

**Figure 9:**
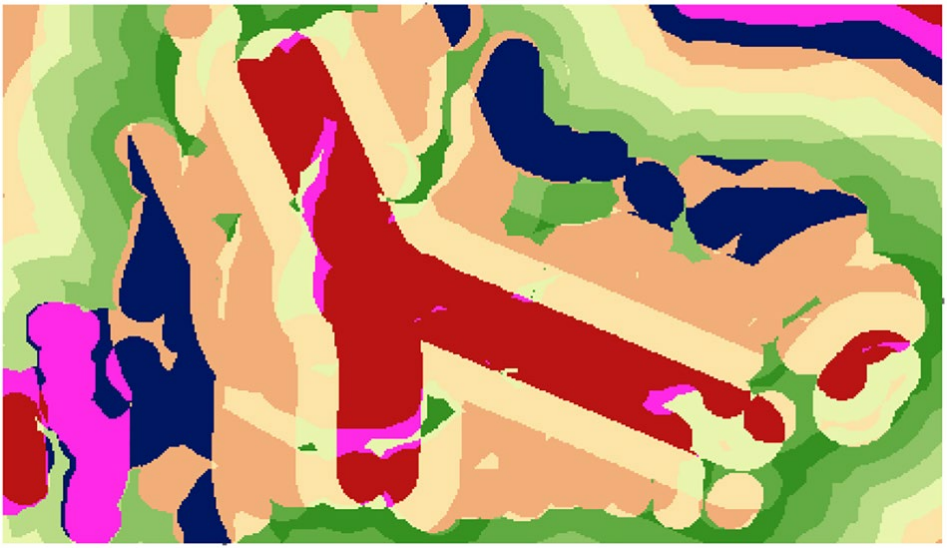
Weighted overlay for layers.

Several layers were overlaid as the form of raster to collect it in one layer through the weighted score. This layer was collected between roads and canal which represented by amusement scale according important it (roads 50% and canal 50%). The final results were: Weights range from 0 to 10 and 0 means unsuitable while 10 means suitable as Fig. 9.

**Figure 10:**
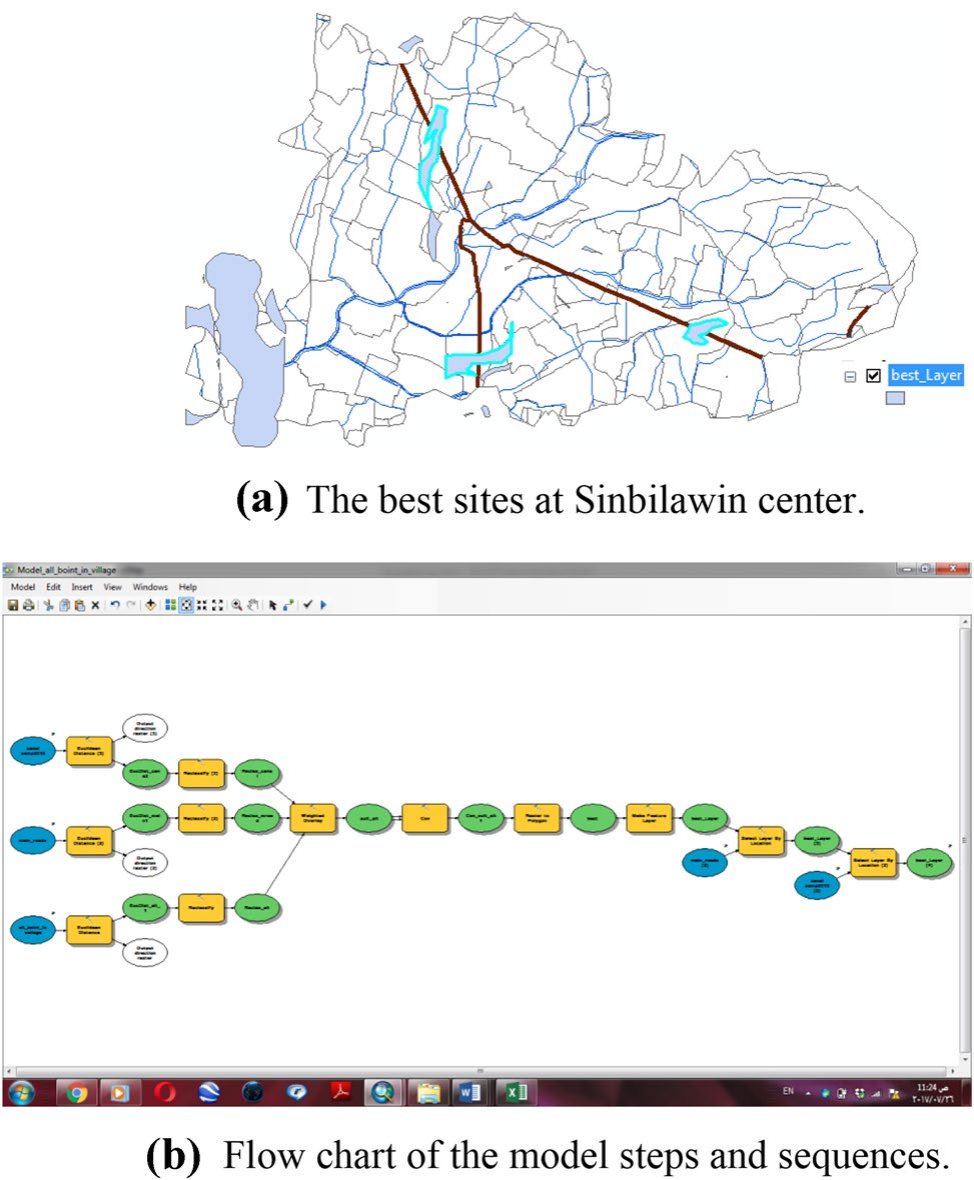
(**a**) The best sites at Sinbilawin center. (**b**) Flow chart of the model steps and sequences.

Finally, three sites from 49 sites as shown in Table 1 were selected for management of agricultural waste as Fig. 10a,b.

**Table 1:** The three sites in Sinbilawin center.

No. of unit	Total weight of rice straw, ton	Name of village
1	30,377.892	Shopra Kopala
2	22,354.61535	Abo El kramet
3	6617.94938	Borg Nor Elarab

#### Third scenario

From the result of the previous scenario was designed a new layer which included the best sites for collecting rice straw in all village.

**Figure 11:**
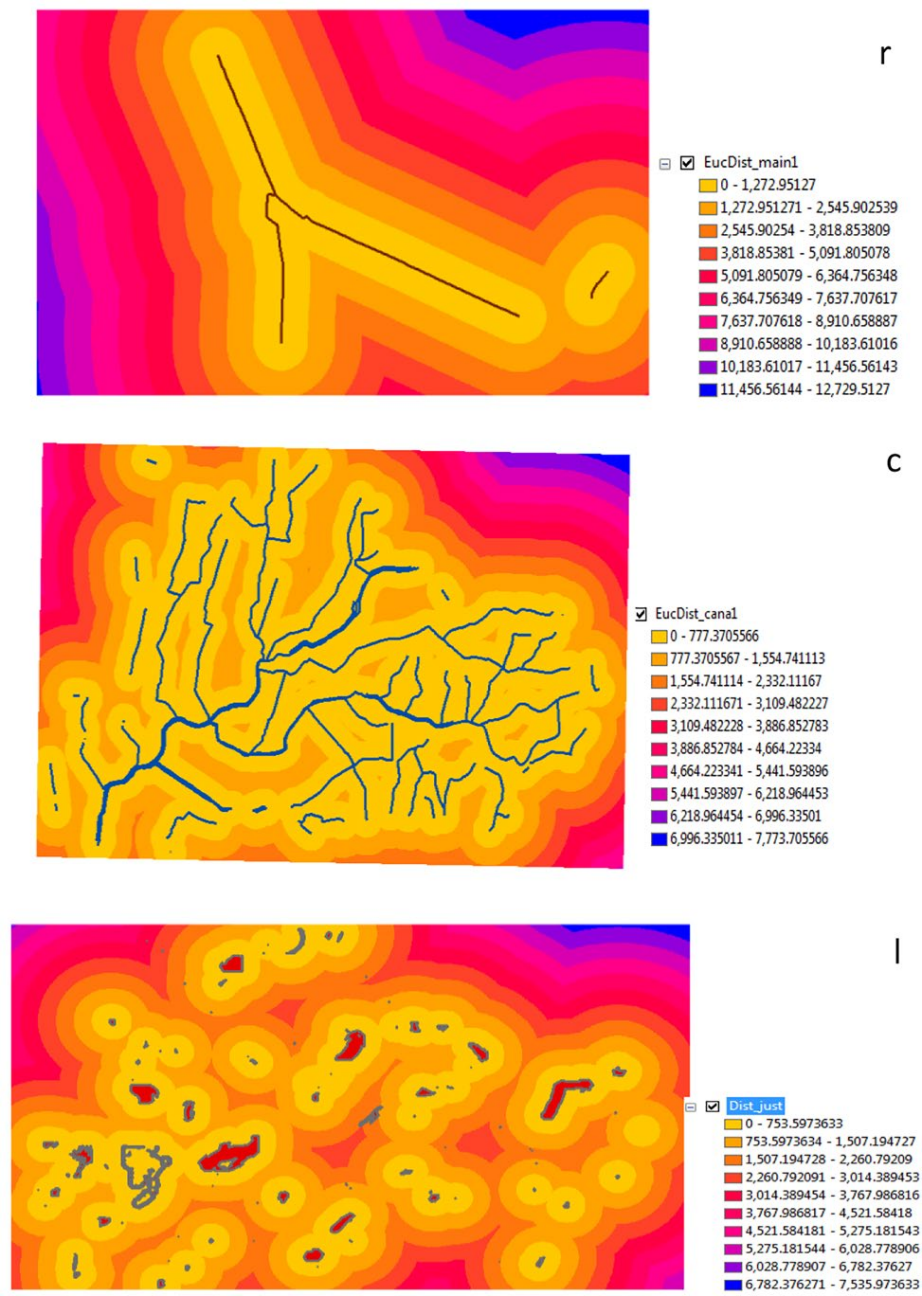
Reclassify: (r) for main road (c) for main canals (l) for the best location in all villages.

From this step was resulted in three categories for main roads, canals and new layer as shown in Fig. 11.

**Figure 12:**
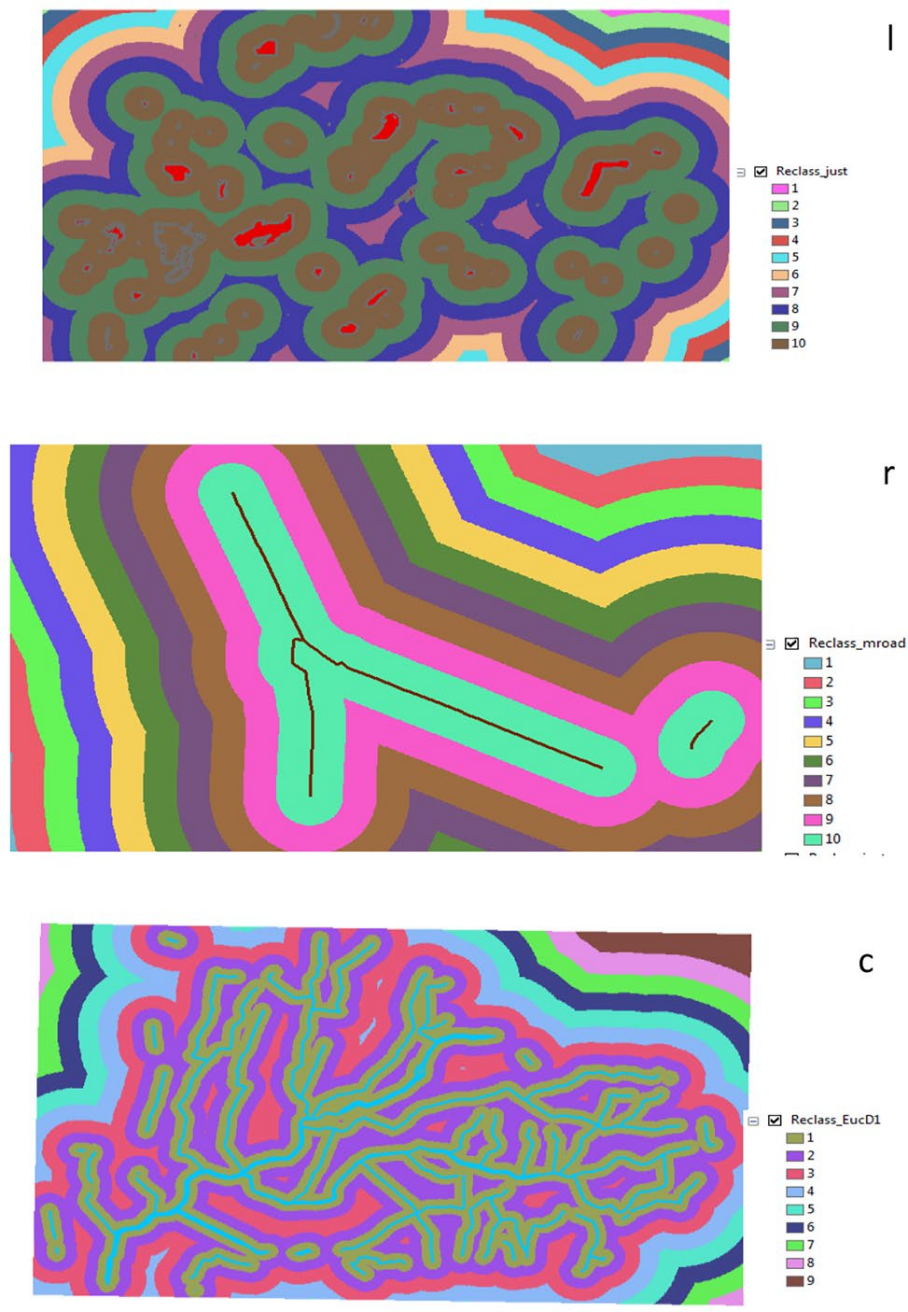
Reclassify: (l) for the best location in all villages (r) for main roads (c) for canals.

Reclassification was used function for canals, main roads and the best location (new layer) in all villages as Fig. 12.

**Figure 13:**
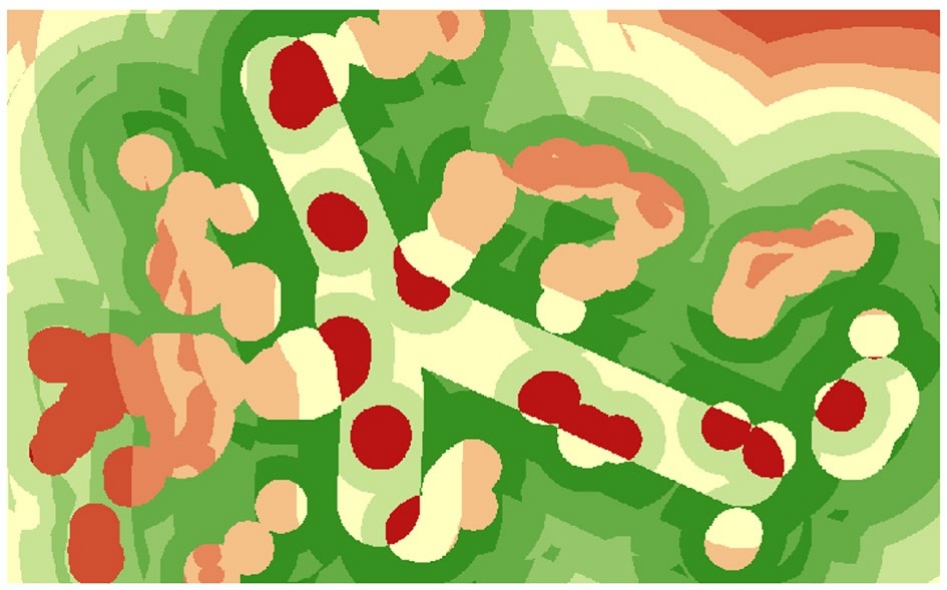
Weighted overlay.

Several layers were overlaid as the form of raster to collect it in one layer through the weighted score. This layer was collected between roads and canal which represented by amusement scale according important it (roads 50% and canal 50%). The final results were: Weights range from 0 to 10 and 0 means unsuitable while 10 means suitable as Fig. 13.

**Figure 14:**
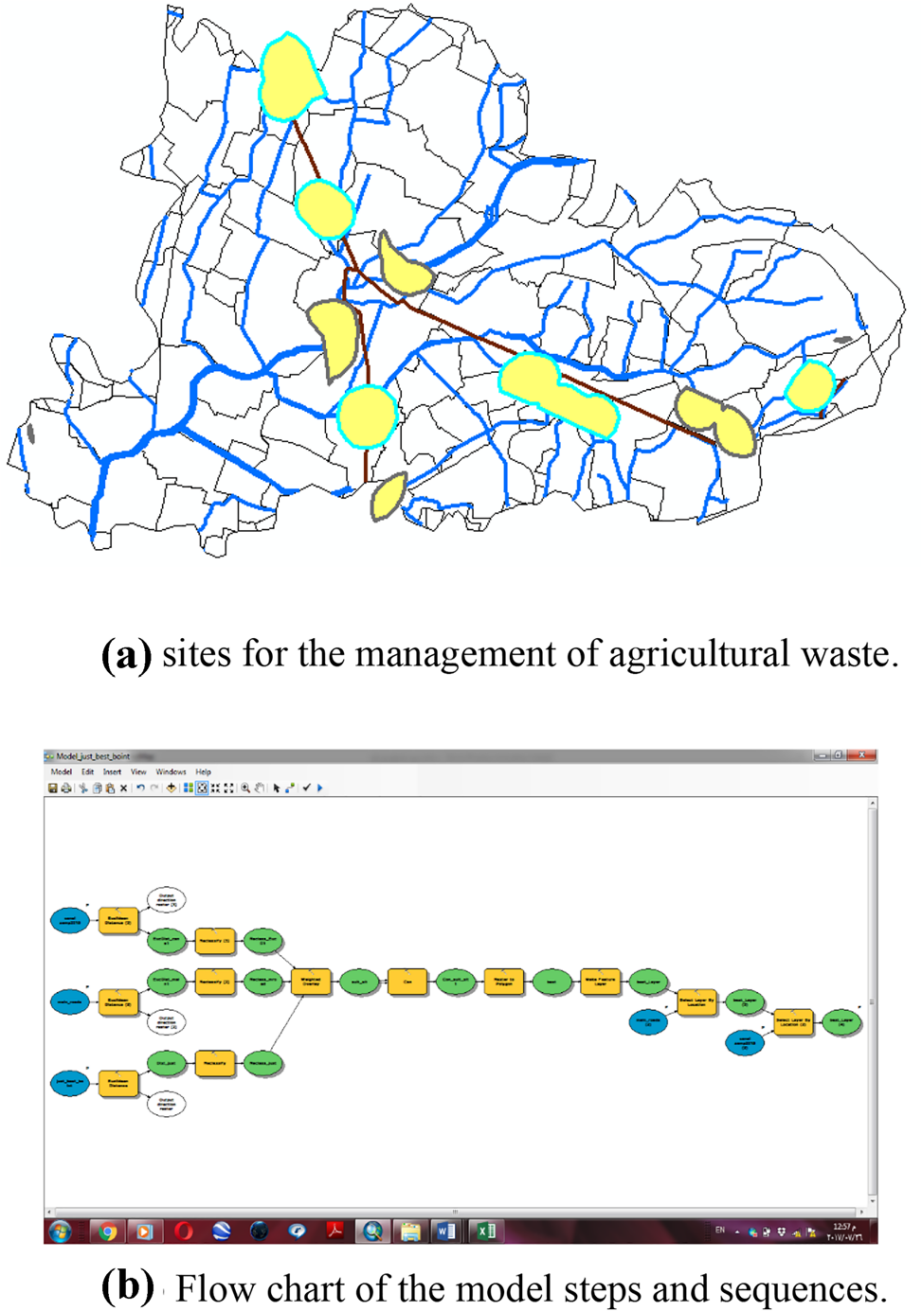
**(a)** Sites for the management of agricultural waste. **(b)** Flow chart of the model steps and sequences.

Finally, five sites from 11 sites as shown in Table 2 were selected for the management of rice straw which located in Sinbilawin center as shown in Fig. 14a,b.

**Table 2:** The five sites in Sinbilawin center.

No. of units	Total weight of rice straw (ton)	Name of village
1	6267.969469	El-Zoraky
2	23,751.23794	Shopra Kopala
3	14,240.48433	Kafr El- Shorafa
4	5303.274464	El Hagiza
5	6617.94938	Borg Nor Elarab

### Transportation cost

In the first scenario the assembly sites were located between more than village. Transportation costs were difficult to calculate because of the difficulty to access a network of documented roads from satellite maps to use it with the GIS program.

Table 3 shows the total transportation costs in the second and third scenarios were 987,308.86 and 826,956.43 L.E. respectively. The average transport costs per ton were 17 and 14 L.E / ton for the second and third scenarios, respectively, from internal assembly sites in each village to the main assembly sites. Also, the results indicate that the total length of roads were 817.62 and 615.65 km for the second and third scenarios, respectively.

**Table 3:** Compare between three scenarios of modeling.

Scenario	Number of collecting sites from modeling	Total length of roads (km)	Total cost for transporting (E.L)	Cost for ton (L.E/ton)
First	40	–	–	–
Second	3	817.6	987,308.9	17
Third	5	615.7	826,956.4	14

From the comparison between the three scenarios, the third scenario was the best one because the total length of roads (that used for transporting the rice straw from field to the collection sites) was less than another two scenarios.

## Conclusions and further recommendations

Geographic Information Systems (GIS) was used to determine the best site to collect the residues in order to reduce time and costs. To achieve these three scenarios were studied to reach the best collection sites for recycling rice straw in Sinbilawin center as follow: First scenario the modeling was ran on Sinbilawin center as the whole unit, second scenario the modeling was ran on each village and third scenario the modeling was ran on each best site in each village. Also, total cost for collecting rice straw was studied. From this study we conclude that transportation costs in the first scenario were difficult to calculate because of the difficulty to access a network of documented roads from satellite maps to use it with the GIS program, meanwhile, the total internal transport costs were 987,308.86 and 826,966.43 L.E for second and third scenarios, respectively. The average transport costs per ton were 17 and 14 L.E/ton for the second and third scenarios, respectively. Also, the total lengths of roads were 817.62 and 615.65 km for the second and third scenarios, respectively. Based on the results of this study GIS became effective tools in determining the optimum procedures of dealing with the agricultural wastes and its management. Further studies on utilization of information technology tools should be carried out.

## Data Availability

The datasets used and/or analyzed during the current study available from the corresponding author on reasonable request.
